# Investigation of slow release of urea from biodegradable single- and double-layered hollow nanofibre yarns

**DOI:** 10.1038/s41598-020-76395-6

**Published:** 2020-11-12

**Authors:** Leila Javazmi, Tobias Low, Gavin Ash, Anthony Young

**Affiliations:** 1 School of Mechanical and Mechatronic Engineering, University of Southern Queensland, Toowoomba, Australia; 2 Centre for Crop Health, University of Southern Queensland, Toowoomba, Australia; 3 School of Agriculture and Food Sciences, The University of Queensland, Brisbane, Australia

**Keywords:** Structural properties, Structural properties

## Abstract

Urea is the most common form of nitrogenous fertiliser. Recently, research has focused on the development of delivery systems to prolong fertiliser release and prevent fertiliser loss through leaching and volatilization. This study investigates and compares single- and double-layered hollow nanofibrous yarns as novel delivery systems to encapsulate and release urea. Single-layered hollow poly l-lactic acid (PLLA) nanofibre yarns loaded with urea fertiliser were fabricated using a customized electrospinning. Double-layered hollow nanofibre yarns were produced by electrospinning polyhydroxybutyrate (PHB) nanofibres as an outer layer, with urea-impregnated PLLA nanofibres as the inner layer. Scanning electron microscopy (SEM) with an energy-dispersive spectroscopy (EDS) was used to characterize the morphology of hollow electrospun nanofibre yarns. A total nitrogen instrument (TNM-1) was used to study the urea release from single- and double-layered hollow nanofibres yarn in water. A Carbon:Nitrogen (CN) elemental analyser determined encapsulated nitrogen in PLLA nanofibres samples. Results indicated that urea-impregnated double-layered hollow nanofibre yarns significantly started nitrogen releasing at much lower amount during first 12 h compared to single-layered hollow nanofibre yarns (P value = 0.000). In conclusion, double-layered hollow nanofibre yarn has potential as an effective alternative to current methods for the slow release of fertilisers and other plant-required chemicals.

## Introduction

Nitrogenous fertilisers improve crop yield and quality by promoting plant growth^1^. Among nitrogen fertilisers, urea is commonly preferred as it is cost effective and has a high nitrogen content of 46% by weight. However, as a neutral organic molecule, urea is not readily absorbed by charged soil particles and can volatilize before hydrolysis is achieved^2^. This results in only a fraction of applied urea nitrogen being absorbed by plants, which may result in large quantities of urea being lost in agricultural runoff, contributing to groundwater pollution^3^. Therefore, the application of surplus nitrogen fertiliser is routine across many agricultural industries to ensure that at least nitrogen use is partially effective. The quantity applied may increase further to compensate for losses through volatilization, denitrification, and leaching which may result in further serious environmental hazards^4^. While some examples of losses greater than 40% of applied urea exist, most investigations report losses of approximately 10%^5^. Consequently, the use of slow- or controlled-release fertilisers has been suggested to improve the efficacy of nitrogen fertilisers and overcome these problems^6^. In parallel to this, researchers have aimed to develop systems which control fertiliser release at low cost and minimise soil contamination, while using biodegradable, inexpensive and readily available material^5^.

Nanofibre electrospinning techniques have attracted interest as versatile, low cost techniques to manufacture sub-micron fibres and nanofibres from polymer solutions or polymer melts^7^. Nanofibre-based structures represent novel materials that can encapsulate and release molecules, as well as biological cells, for applications in agricultural, medical and engineering fields^8^.

The advent of composite hollow nanostructures adds a new dimension to nanofibre applications. These have extended the impact of particles by coupling their functionality with the feasible processability of synthetic polymers^9^. Furthermore, such hollow nanostructures have much higher surface area to volume ratios compared to their solid counterparts of the same dimensions, a quality which is beneficial when adsorption or storage of chemicals or charges are required^10^. For example, they include electrospun webs that offer control over pore volume and distribution that facilitates dynamic release of target molecules. The use of nanofibres to encapsulate agrichemicals may also allow different chemical additives to be used together through separate nanofibres, and can prolong agricultural additive release by several months^4,5,11–13^.

To date, few methods have been proposed to obtain hollow yarns by using the electrospinning process. This unique hollow yarn structure brings together some advantages including high volume and low weight of electrospun nanofibres, combined with the large surface areas of three-dimensional nanofibrous architectures. One such method reported by Bhargava in 2007 used a rotating metal rod attached to a metal hemisphere at one end to produce hollow nanofibre yarn. A hollow core-sheath yarn using two different polymer solutions was electrospun by this method. By subsequently removing the inner core yarn, a hollow nanofibre yarn was obtained^14^. Following this work, a modified electrospinning apparatus to fabricate uniaxial aligned nanofibre yarns was introduced by Wu & Qin^15^ . In a recent study, Javazmi et al. (2014) applied a two-nozzle conjugated electrospinning method to fabricate a core-sheath yarn from nanofibres of polyester as the sheath and polyvinyl alcohol (PVA) multifilament as the core of the yarn. Subsequent dissolution of the core yarn in hot water produced a hollow nanofibre yarn^16^. Fakhrali et al. (2014) developed a novel method of fabricating core-sheath nanofibre yarns. This core-sheath nanofibre consisted of PVA nanofibres as the core and nanofibres of nylon 6 as the sheath. Such yarn has potential applications in various fields such as loading drugs into a PVA solution as a core with variable portions^17^.

To the best knowledge of the author, no research study has attempted to investigate and compare single- and double-layered hollow nanofibrous yarn as delivery systems to encapsulate and release chemical additives of agriculture. This research aimed to assess the capability of a novel concept to encapsulate urea molecules within double-layered hollow nanofibrous matrices resulting in prolonged urea release rate compared to single-layered ones.

## Results and discussion

**Figure 1:**
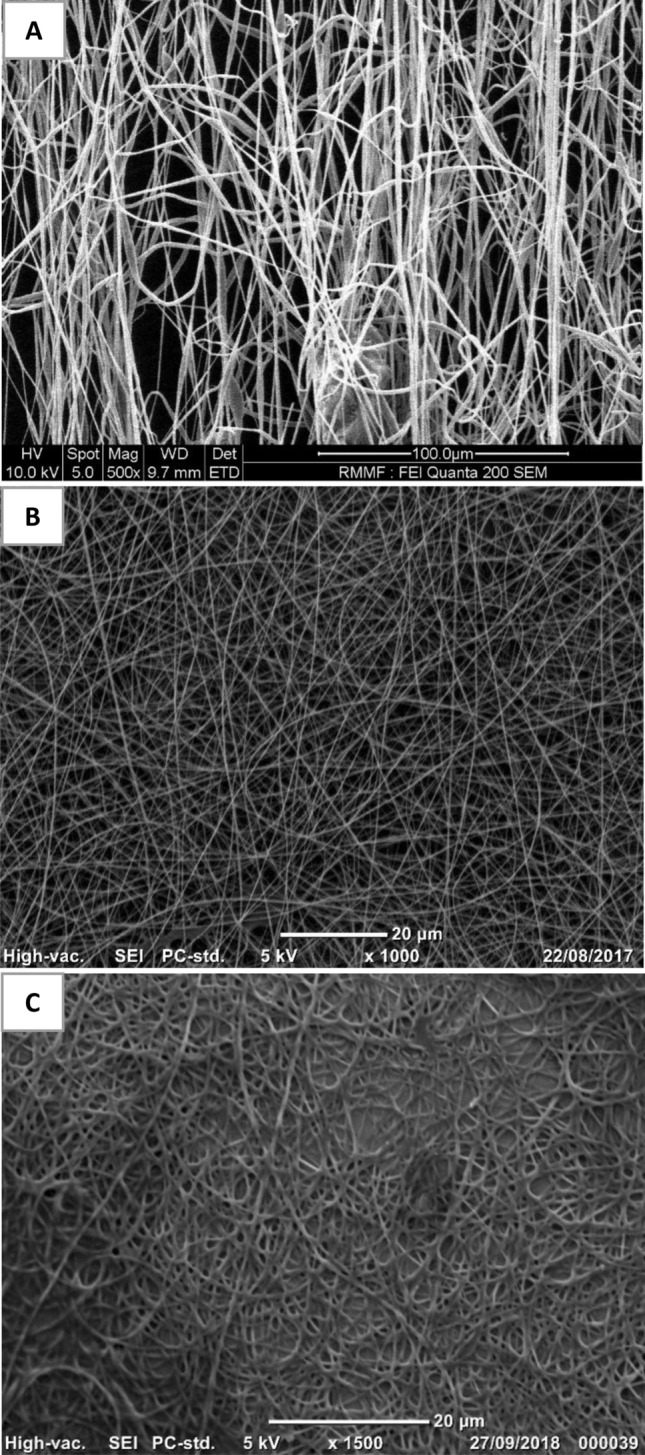
SEM micrographs of nanofibres produced during this study. **(A)** 5% PLLA electrospun nanofibres loaded with 20% urea, **(B)** 10% PVA electrospun nanofibres and **(C)** 7% PHB electrospun nanofibres.

PVA, PLLA loading urea, and PHB electrospun nanofibres were bead free and exhibited good uniformity (Fig. 1; Table 1). Uniformly produced electrospun nanofibres indicated that electrospinning parameters were optimized accurately^18–21^.

**Table 1:** Diameters of PLLA, PVA, and PHB electrospun nanofibres at specific polymeric solutions.

Solution type	Concentration (%) (w/w) of polymeric solution (%)	Nanofibre diameter (nm)
PLLA loading 20% urea in CF:acetone (3:1)	5	710.00 ± 262.70
PVA in distilled water	10	242.14 ± 22.20
PHB in DMF:CF (3:7)	7	417.58 ± 63.80

**Figure 2:**
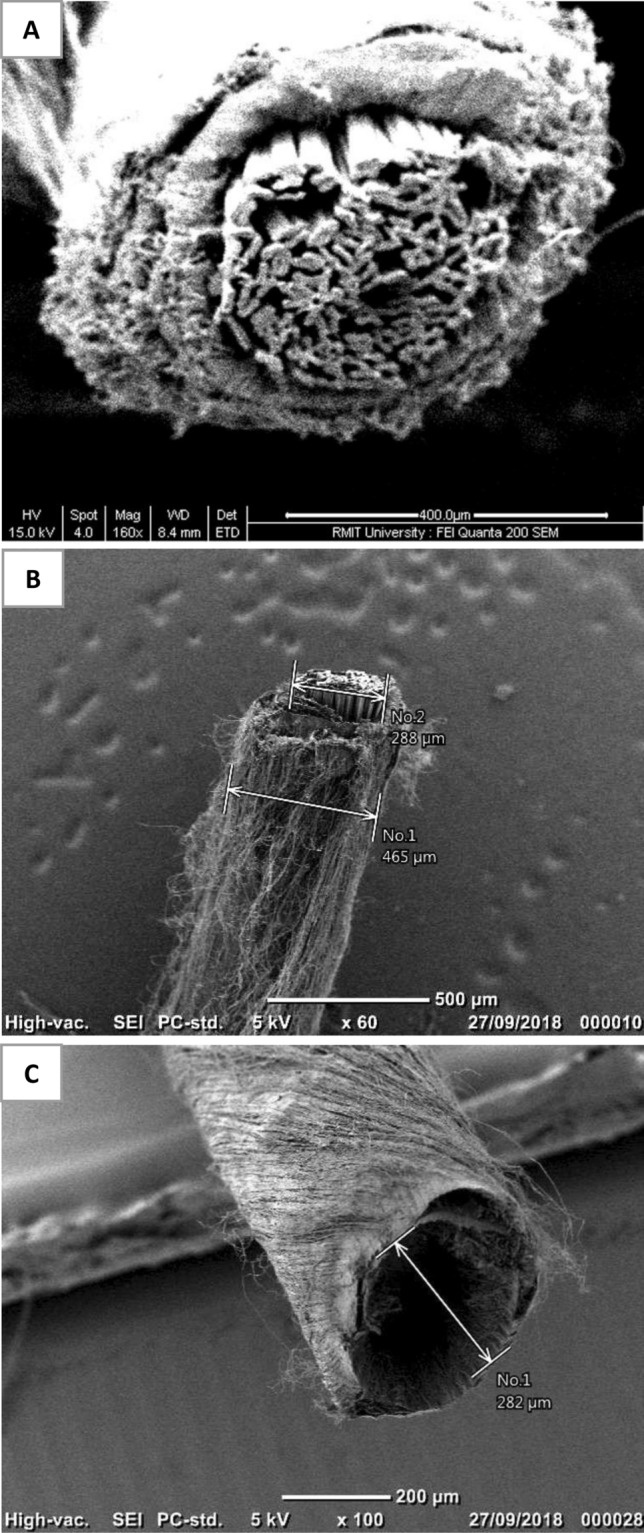
Scanning electron microscopy images of: **(A)** the cross section of quad-layered electrospun nanofibre yarn **(B)** the surface area of quad-layered electrospun nanofibre yarn, and **(C)** the cross-section area of hollow double-layered electrospun nanofibre yarn.

The diameters of the quad-layered yarn core section and double-layered hollow nanofibre yarns were 288 ± 3 and 282 ± 2 µm, respectively (Fig. 2). The decrease in the diameter of the core space is due to placement of core-sheath nanofibre yarn in boiling water for one minute to dissolve the PVA nanofibres that led to lateral shrinkage of the hollow nanofibre yarn. Also, the process decreased the air gap between nanofibres resulting in nanofibre layers sticking together^17^.

**Figure 3:**
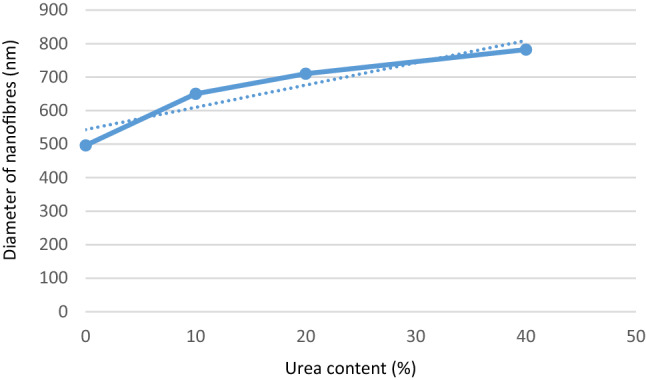
The relationship between the electrospun PLLA- urea nanofibre diameter and the urea content.

**Figure 4:**
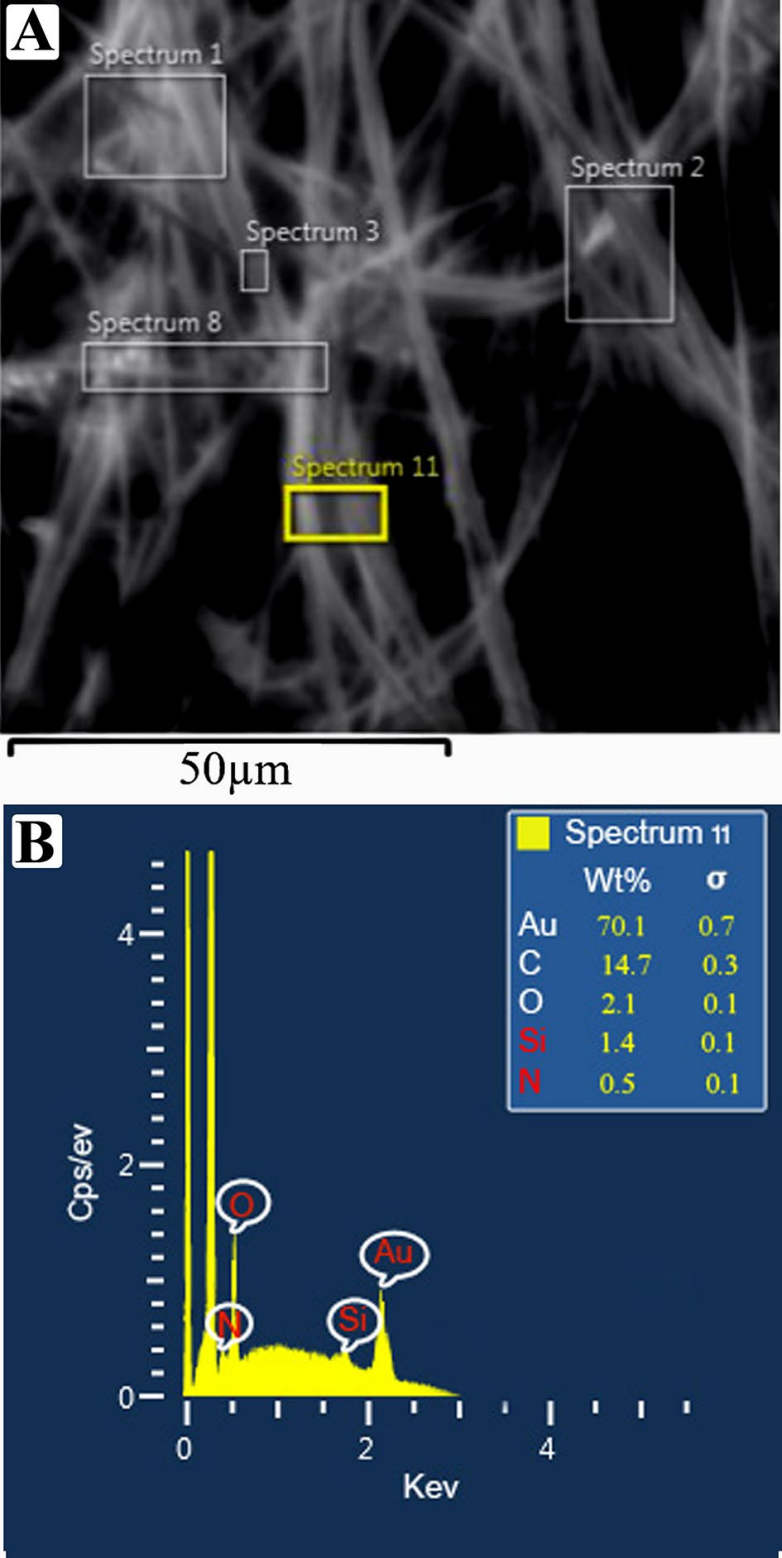
**(A)** SEM micrograph of PLLA nanofibre containing 20% urea, and **(B)** a typical EDS analysis of PLLA nanofibre containing 20% urea.

Electrospun nanofibre diameter increased with increasing urea concentration from 496 to 782 nm (Fig. 3). This was expected as higher viscosity tends to lead to higher diameter electrospun nanofibres^22^. A typical SEM micrograph and EDS analysis of PLLA nanofibre containing 20% urea is illustrated in Fig. 4A,B. The EDS shows the amount of nitrogen element in spectrum 11 based on (w/w) % to PLLA mass amount. From the analysis, it is evident that nitrogen, which is equal to 46% of urea mass, was successfully loaded into all PLLA nanofibre layers. The average percentage amount of main identified elements in spectrums from 1 to 11 is shown in Table 2.

**Table 2:** Average percentage of identified elements in PLLA nanofibres shown in EDS spectrums as above.

Statistics	C (%)	O (%)	N (%)
Max Min Average Standard deviation	15.80 9.80 13.43 2.47	2.50 1.50 1.87 0.31	0.50 0.00 0.13 0.13

**Figure 5:**
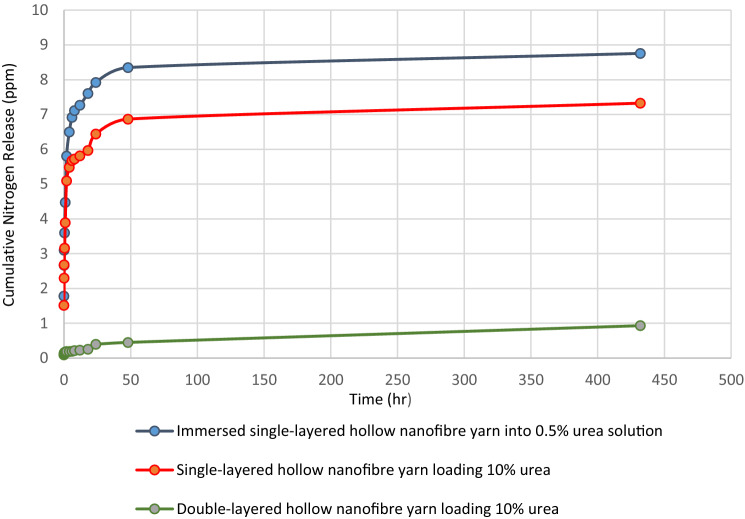
Cumulative nitrogen release from immersed nanofibre yarn in 0.5% urea solution and single-, double-layer hollow nanofibre yarns containing 10% urea.

One-way ANOVA analysis showed nitrogen release commenced at a higher rate as urea concentration increased from 10 to 40% into both single-layer and double-layer hollow nanofibre yarn structures. Thus, as may be expected, by increasing the percentage of urea loaded into nanofibres, nitrogen release rate increased. Double-layered nanofibres containing 10% urea showed a significant decrease (P value = 0.003 and 0.000) in release rate compared with double-layered samples loading 20% and 40% urea, respectively which demonstrated 10% loaded urea into nanofibres as the optimum load. The cumulative nitrogen release for double-layer hollow nanofibre yarn loading 10% urea was approximately 24% of total release while control sample and single-layer hollow nanofibre yarn containing 10% had achieved over 82% and 79% release, respectively, during first 12 h (Fig. 5). Double-layer hollow nanofibre yarn structures containing 10% urea release nitrogen at much lower rate compared to single-layer hollow nanofibre yarn encapsulating urea by either electrospinning fabrication or immersing deposition. PHB nanofibrous layer as a physical barrier around PLLA nanofibres in double-layer hollow yarn delayed urea release loaded in PLLA nanofibres. The release mechanism of nitrogen from PLLA nanofibre matrix followed both diffusion and degradation kinetic release in water^23^.

Table 3 shows nitrogen content of urea- impregnated PLLA nanofibre yarns at concentrations of 0%, 10%, 20%, and 40% by CN analyser. By increasing urea content from 0 to 40%, the percentage of nitrogen increased significantly (P value = 0.03) from 0.06% to 5.23%, respectively, which indicated that nitrogen was encapsulated successfully.

**Table 3:** CN analysis of PLLA nanofibres loading using different percentages of urea.

Sample	Weight (mg)	Nitrogen (%)	Carbon (%)
PLLA nanofibre loaded with 0% urea	64.02	0.06	53.66
PLLA nanofibre loaded with 10% urea	22.24	1.44	50.92
PLLA nanofibre loaded with 20% urea	30.10	2.56	51.24
PLLA nanofibre loaded with 40% urea	41.72	5.23	51.60
Pure Urea	63.60	46.32	20.12
*P value for nitrogen percentage of different loaded urea	0.03 ≤ 0.05		

*P value probability value.

If P value ≤ 0.05, there is significant difference between data.

## Conclusion

Single- and double-layer hollow nanofibre yarn structures were fabricated successfully and loaded with urea using the electrospinning technique. Increased urea concentration produced larger nanofibre diameters. The urea concentration did not affect the nitrogen release rate efficiency within single- or double-layer hollow nanofibre yarns significantly. Moreover, double-layer hollow nanofibre yarns containing urea exhibited a significant reduction in the release amount of nitrogen compared to single-layered nanofibre yarns. Energy-dispersive detector analysis of PLLA nanofibres loading urea illustrated that nitrogen element was encapsulated into PLLA nanofibrous structure. This work has demonstrated that double-layer hollow PLLA nanofibre yarn containing urea may be an effective carrier to control the release of urea fertiliser for future research in agriculture field.

## Methods

### Materials

PVA with average molecular weights (MW) ranging from 89,000 to 98,000 g/mol (Product Number: 341584), PHB (Product Number: 363502), and solvents, *N*-dimethylformamide (DMF); Reagent Plus^®^,  ≥ 99%, chloroform (CF); anhydrous,  ≥ 99%; and acetone (Ace) for HPLC,  ≥ 99.8% were obtained from Sigma Aldrich. PLLA with a MW of 282,000 g/mol was obtained from Vorina Biomaterials, Ireland (CAS Number: 33135-50-1). Urea (% N: P: K; 46:0:0) from Richgro Garden Products was used in all experiments. A 400/112 denier PVA multifilament yarn was obtained from Dongguan Cocou Textile Materials Company in China.

### Preparation of PVA, PLLA and PHB solutions

An aqueous PVA solution with a concentration of 10% (w/w) was prepared by adding Milli-Q water to PVA powder and heating to 80 °C with stirring for 20 min. A PLLA solution with a concentration of 5% (w/w) in chloroform: acetone (3:1 v/v) was prepared and mixed with 10%, 20%, and 40% (w/w) urea powder relative to the total weight of dissolved PLLA. A PHB polymeric solution in DMF: CF (30:70 v/v) solvent was used at a concentration of 7% (w/w).

### Electrospinning apparatus

**Figure 6:**
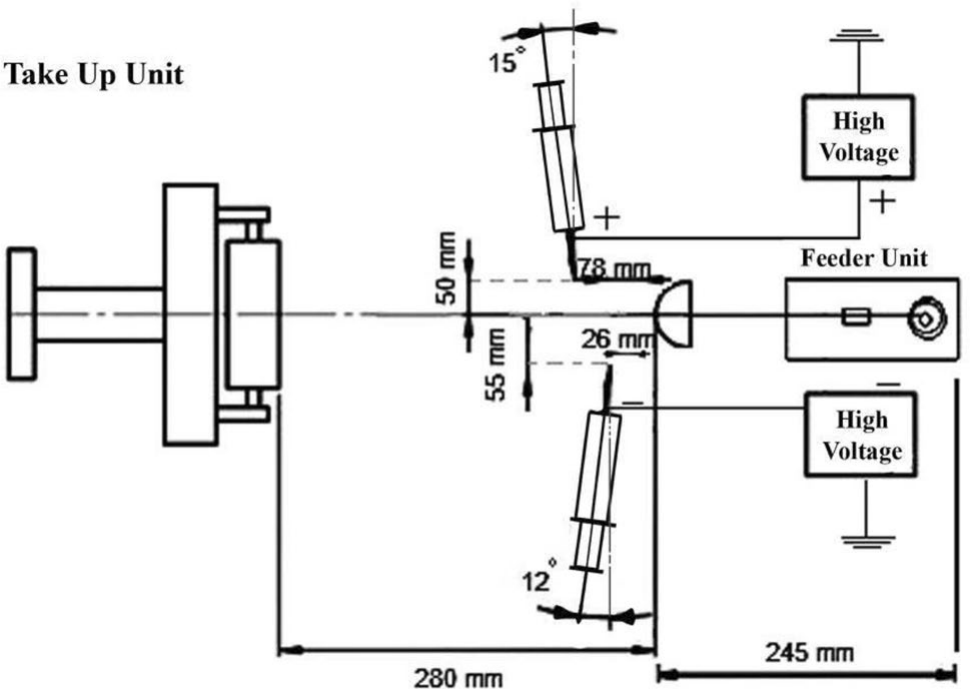
Schematic of custom electrospinning apparatus used in this study.

To produce single- and double-layered hollow nanofibrous yarn, a customised electrospinning apparatus with two different sets of electrospinning parameters was used in the first and second stages. The schematic diagram of the modified apparatus (Fig. 6) displays the three main sections that include a feeder unit, middle and a take up unit. The feeder unit was a negative feeder system that fed a PVA multifilament core yarn from a bobbin using take up unit tension. A friction disc tensioner was attached to the bobbin to control the PVA multifilament tension. Electrospinning was conducted in the middle unit, which consisted of two high voltage power supplies (HV), model 73030 DC input 30 kV@1 mA, from Genvolt, Ireland, Two NE-300 ‘Just Infusion’ syringe pumps from New Era Pump Systems Company, Australia, and a rotating conductive hemisphere with a hollow metal rod attached. The take up unit, that drew electrospun nanofibre yarn at a speed of 1.62 cm/min, consisted of a 5 cm × 8 cm cylindrical roller, and a stepper motor to control the speed of uptake using an ATMEGA328P Pro Mini 328 Mini 16 MHz microcontroller.

Positively and negatively charged electrodes were connected to the needles with angles of 15º and 12º, respectively. During the two different stages of the electrospinning process, the angular placement of the needles in the apparatus mentioned above facilitated fabrication of two distinctive sheaths of nanofibres surrounding the core yarn. The positively charged needle was spaced 78 mm horizontally, and 50 mm vertically from the hemispherical collector, while the negatively charged needle was positioned 26 mm horizontally and 55 mm vertically from the hemispherical collector.

The aluminium-covered hemispherical collector, with neutral charge and 80 mm diameter, was located 280 mm and 245 mm away from the take-up unit and feeder unit respectively. Angular velocity of the collector was 32 rpm and it could rotate both clockwise and anti-clockwise.

During the first stage, single-layered hollow nanofibrous yarn was fabricated. PVA multifilament was fed through the hemispherical collector by the feeder unit, and the take-up roller collected the other end of the folded PVA multifilament at a rate of 1.62 cm/min. The PVA solution with concentrations of 10%, and 5% (w/w) PLLA solutions loaded with urea at concentrations of 0%, 10%, 20%, and 40% were introduced into the electric field at feeding rates of 0.5 mL/h and 1 mL/h respectively. Electrospun nanofibres were formed between two needles with opposite polarities on the rotating hemispherical collector surface. The different electrical potential between the two needles was 21 kV. By this method, the PVA multifilament was firstly coated by PVA nanofibres fed through the negative charged needle and then by PLLA nanofibres loaded with 10% urea formed from the positive charged nozzle.

**Figure 7:**
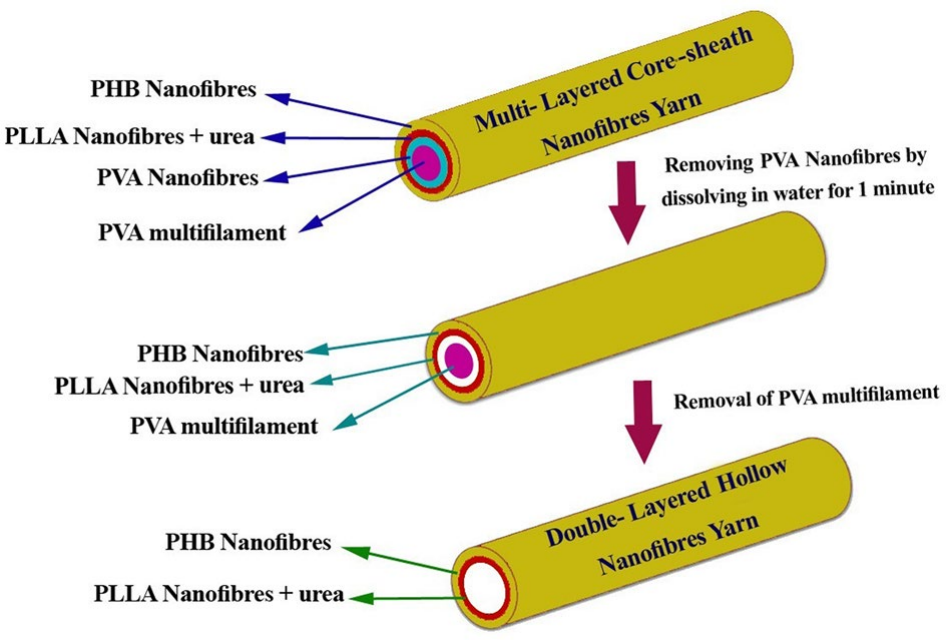
Demonstration of how double-layered hollow nanofibres loading urea are obtained from quad-layered core-sheath nanofibre yarns.

During the second stage, the feeder unit fed the triple layered nanofibre yarn that was achieved from first stage. The same electrospinning apparatus and parameters are applied for the second stage, however both positive and negative needles are directly located 120 mm from each other and 26 mm from the hemisphere both needles with flow rates of 1 mL/h used to inject 7% (w/w) PHB solutions. PVA multifilament coated by PVA and PLLA nanofibres passed through the rotating collector and was covered by a layer of PHB nanofibres. Finally, the take-up roller collected the four distinctively layered nanofibre yarns. Electrospinning of solutions was conducted at 32 °C and ambient humidity. To produce double- layered PLLA/PHB hollow nanofibre yarn, the PVA multifilament core yarn was easily removed after dissolving the PVA nanofibre in water at room temperature for one minute as shown in Fig. 7^24^.

### Characterization

The morphology of electrospun nanofibres, PVA, PLLA loading urea, and PHB nanofibres, and hollow nanofibre yarns was determined by SEM (FEI Quanta 200 ESEM 2002) with attached EDS at RMIT Microscopy and Microanalysis Facility (RMMF) and benchtop SEM (JEOL JCM-6000 PLUS) at the University of Southern Queensland (USQ). Image Java analysis software was used to measure PVA, PLLA and PHB nanofibre diameters from SEM images at magnifications of 1000 × and 5000 ×.

The single- and double-layered hollow nanofibrous yarns loaded with urea were cut into 6 cm lengths. These pieces were weighed with an analytical balance accurate to ± 0.00001 g and placed in a plastic tube with 20 mL of Milli-Q water to soak for 2 min to dissolve any superficial urea deposited on the surface. In addition, a single-layered hollow nanofibre yarn without loaded urea immersed in 0.5% urea solution for 24 h was selected as a control sample. Subsequently, each sample was immersed in 20 mL Milli-Q water in a 50 mL plastic tube and placed in a shaker at 70 RPM and 30 °C ^4^. The samples were then removed from the shaker and immersed in a tube containing 20 mL of fresh Milli-Q water for different periods, ranging from 0 to 440 h. A Shimadzu TNM-1 total nitrogen instrument was used to measure nitrogen release from these nanofibre mats. Cumulative nitrogen release of each sample was calculated for the selected period and reported as % nitrogen. Three replicates were produced for all samples and results were reported as the average ± one standard deviation.

Nitrogen content of urea-impregnated nanofibres yarns was analysed by a CN analyser (LECO CN626 analyser, Michigan, USA). The single- and double-layered hollow nanofibrous yarn samples were weighed in tin containers and then shaped to little round balls. Tin was important for the correct combustion in the elemental analyser. The tin cups were then dropped into a tube where in the presence of external oxygen flash combustion occurred at a temperature of 1800 °C.
